# The time cost of physiologically ineffective intravenous fluids in the emergency department: an observational pilot study employing wearable Doppler ultrasound

**DOI:** 10.1186/s40560-023-00655-6

**Published:** 2023-02-15

**Authors:** Jon-Émile S. Kenny, Stanley O. Gibbs, Delaney Johnston, Zhen Yang, Lisa M. Hofer, Mai Elfarnawany, Joseph K. Eibl, Amanda Johnson, Anthony J. Buecker, Vivian C. Lau, Benjamin O. Kemp

**Affiliations:** 1 Health Sciences North Research Institute, 56 Walford Road, Sudbury, ON P3E2H3 Canada; 2 Flosonics Medical 325 W. Front Street, Toronto, ON M5V2Y1 Canada; 3 Northern Ontario School of Medicine, 935 Ramsey Lake Road, Sudbury, ON P3E2C6 Canada; 4 OSF Saint Francis Medical Center, 530 NE Glen Oak Ave, Peoria, IL 61637 USA

**Keywords:** Doppler ultrasound, Carotid artery, Fluid responsiveness, Fluid refractory, Quality improvement, Personalized medicine, Wearable technology

## Abstract

**Background:**

Little data exist on the time spent by emergency department (ED) personnel providing intravenous (IV) fluid to ‘responsive’ versus ‘unresponsive’ patients.

**Methods:**

A prospective, convenience sample of adult ED patients was studied; patients were enrolled if preload expansion was indicated for any reason. Using a novel, wireless, wearable ultrasound, carotid artery Doppler was obtained before and throughout a preload challenge (PC) prior to each bag of ordered IV fluid. The treating clinician was blinded to the results of the ultrasound. IV fluid was deemed ‘effective’ or ‘ineffective’ based on the greatest change in carotid artery corrected flow time (ccFT_∆_) during the PC. The duration, in minutes, of each bag of IV fluid administered was recorded.

**Results:**

53 patients were recruited and 2 excluded for Doppler artifact. There were 86 total PCs included in the investigation comprising 81.7 L of administered IV fluid. 19,667 carotid Doppler cardiac cycles were analyzed. Using ccFT_∆_ ≥  + 7 ms to discriminate ‘physiologically effective’ from ‘ineffective’ IV fluid, we observed that 54 PCs (63%) were ‘effective’, comprising 51.7 L of IV fluid, whereas, 32 (37%) were ‘ineffective’ comprising 30 L of IV fluid. 29.75 total hours across all 51 patients were spent in the ED providing IV fluids categorized as ‘ineffective.’

**Conclusions:**

We report the largest-known carotid artery Doppler analysis (i.e., roughly 20,000 cardiac cycles) in ED patients requiring IV fluid expansion. A clinically significant amount of time was spent providing physiologically ineffective IV fluid. This may represent an avenue to improve ED care efficiency.

## Introduction

As a concept, fluid responsiveness has grown and evolved over the last 20 years [1–3]. At its core is the notion that intravenous (IV) fluid has an intended effect, that is, to increase stroke volume (SV) [4, 5]. This cause-and-effect relationship between cardiac input (i.e., preload, IV fluids) and SV is described by the ‘Starling’, or ‘cardiac function’, curve [6, 7]. Critically, during acute illness many patients flatten their cardiac function curve such that augmenting preload with IV fluid does not have the intended effect of improving SV [8, 9]. Therefore, without measuring blood flow change in response to IV fluid, the desired outcome of administering preload is uncertain.

Despite the aforementioned physiological rationale for individualizing IV fluid therapy, there is little and conflicting data supporting better outcome with this paradigm, especially in the emergency department (ED) [10]. For example, a meta-analysis of goal-directed fluid therapy in the operating room revealed that flow-guided resuscitation improved patient outcomes [11], though when restricted to septic, critically ill patients, no clear differences were observed [12]. Two recent evaluations of septic patients in the intensive care unit (ICU) reported that flow-guided IV fluid resuscitation diminished fluid administration, complications secondary to overload (e.g., mechanical ventilation time, renal replacement therapy) and cost as compared to standard care [13, 14]. These findings led some authorities to reframe testing for ‘fluid responsiveness’ as, instead, evaluating for a ‘fluid refractory’ state [15]. Conversely, two trials in septic ED patients found that patients managed by ‘fluid responsiveness’ assessments received *more* IV fluids without any patient-centered improvement [16, 17]. Importantly, however, in both ED studies the resuscitation protocols encouraged IV fluids until fluid responsiveness disappeared—an inappropriate approach [3].

In addition to patient-centered outcomes, another perspective on IV fluid administration is that of resource utilization in the ED. Arguably, unnecessary tests and therapies hinder ED throughput and the provision of physiologically ineffective IV fluid could be considered in this regard. In both ED studies above, there were relatively high initial fluid *unresponsive* rates (i.e., 21% [16] and 53% [17]). Similar fractions were noted early in the FRESH [13] and in ANDROMEDA-SHOCK [18] trials. Moreover, in the critically ill cohort of ANDROMEDA-SHOCK, withholding IV fluid in unresponsive patients did not cause harm [9]. Therefore, early detection of fluid unresponsiveness (i.e., a ‘fluid refractory’ patient) in the ED could save both patients from an arguably ineffective medical therapy and providers from the time spent carrying out an unneeded intervention.

With the above, the STudying the Over-Prescription of IV FLuids via an ObservatiOnal Doppler INvestiGation (STOP-FLOODING) was designed and completed as a pilot study in a community ED. Our primary goal was to quantify the burden of fluid unresponsiveness early in ED care; our secondary goal was to calculate the time spent providing physiologically ineffective IV fluid. These objectives were accomplished using a novel, wireless, wearable Doppler ultrasound system [19]. We have previously shown a strong, linear correlation between the change in the common carotid artery corrected flow time (ccFT_∆_) and stroke volume (SV_∆_) using this device [20, 21]. Furthermore, Barjaktarevic and colleagues found that ccFT_∆_ could accurately detect SV_∆_ in undifferentiated shock [22]. Therefore, to accomplish our primary and secondary goals, we measured the ccFT_∆_ during a preload challenge prior to each bag of IV fluid and with the treating clinician blind to the results of the Doppler ultrasound.

## Materials and methods

A prospective, convenience sample of adult patients presenting to a single community ED in Peoria, Illinois, U.S.A. was studied. The patients were enrolled between February and April 2022. The study was performed in accordance with the ethical standards as laid down in the 1964 Declaration of Helsinki and its later amendments. Patients or their legal representative provided written, informed consent and the study was approved by the Peoria Institutional Review Board (# 1697834-5).

Adult patients were enrolled if the treating clinician determined that IV fluid expansion would be beneficial for any indication. Patients were excluded if they were not at least 18 years old, if they did not provide informed consent, if they were unable to cooperate with a Doppler ultrasound assessment of the carotid artery (e.g., delirium, confusion, excessive phonation, etc.) or if there were anatomical contraindications precluding assessment of at least one carotid artery (e.g., known bilateral carotid stenoses of at least 70%, bilateral internal jugular central lines, c-spine collar, etc.).

**Fig. 1:**
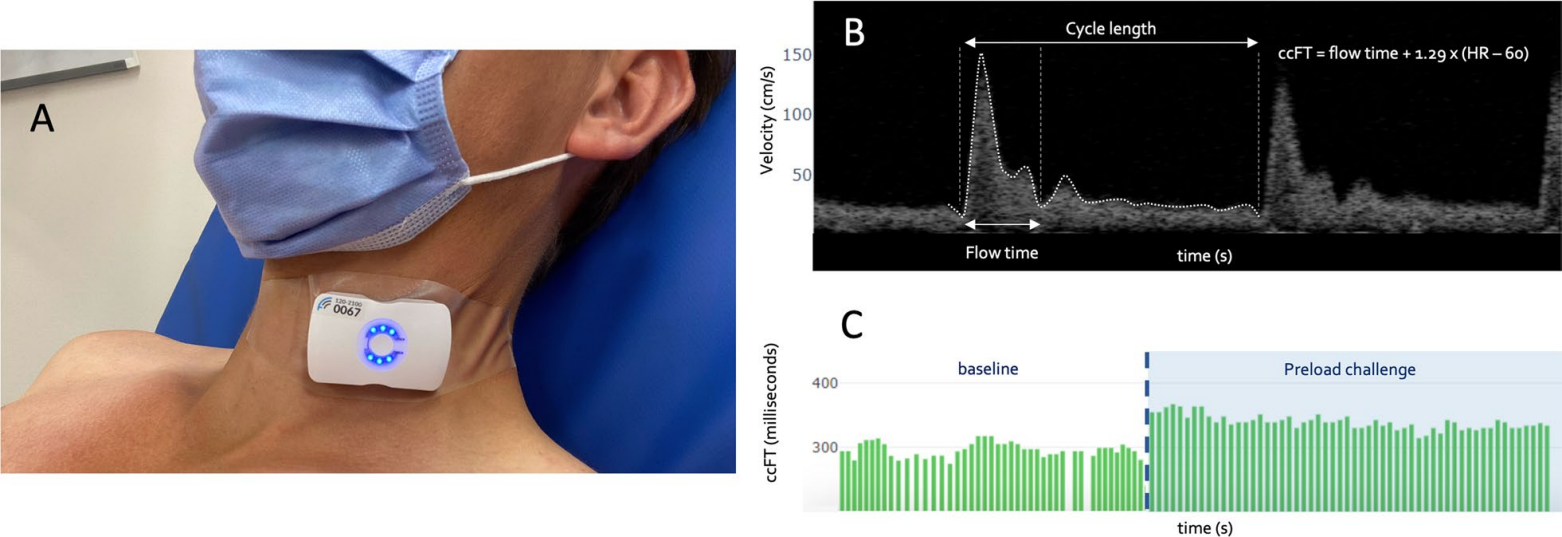
The wearable Doppler ultrasound and its interface. **A** The device on a healthy volunteer. **B** Carotid Doppler spectra showing calculation of carotid corrected flow time (ccFT) by the equation of Wodey. Velocity is on y-axis in centimeters per second (cm/s) and time on x-axis in seconds (s); heart rate (HR) is calculated from cycle length. **C** Example of a preload challenge shown on the graphical user interface of the wearable Doppler. Each green bar represents the ccFT of a single cardiac cycle; the *y*-axis is ccFT in milliseconds and the *x*-axis is time in seconds

Prior to each IV fluid bag of at least 500 mL, 30–60 s of resting carotid Doppler was recorded using a wireless, wearable Doppler ultrasound (Flosonics Medical, Sudbury, Ontario, Canada). The wearable Doppler system automatically traces the maximum velocity envelope of the carotid artery Doppler pulse and calculates the corrected flow time (ccFT) by the equation of Wodey [20–23] (Fig. 1). Immediately thereafter, a preload challenge (PC) was performed. At the discretion of the treating clinician, the PC could be either a rapid fluid challenge (RFC) or a passive leg raise (PLR). The treating clinician was blinded to all measures from the wearable Doppler ultrasound; continuous carotid Doppler was recorded throughout the entirety of the PC only.

The RFC consisted of the first 250 mL of the fluid bag delivered at a rate of at least 100 mL/min [24–26]. This infusion rate was accomplished based on the size of the intravenous catheter through which the fluid was delivered [27]. If pressure was required to achieve an adequate infusion rate, either a standard pressure bag or the LifeFlow device (410 Medical, Durham, North Carolina, U.S.A.) was employed, at the discretion of the treating clinician. The PLR consisted of moving the patient from semi-recumbent baseline to supine with the legs passively raised for at least 90 s, per expert recommendation [28, 29].

The Doppler spectra were analyzed for the absolute and % ccFT_∆_. The number of cardiac cycles averaged before and during preload augmentation was dictated by the coefficient of variation of the ccFT to ensure change could be detected with statistical confidence [30]. The assessment windows showing the largest change between baseline and preload augmentation were considered for analysis.

After completing the PC by either RFC or PLR, IV fluid was infused at the discretion of the treating clinician. For example, if a patient was ordered for 1.5 Liters (L) of IV fluid, 2 PCs were performed, 1 prior to the 1-L bag and 1 prior to the 500-mL bag. If the PCs were accomplished by RFC, the first 250 mL of each bag were infused into the patient at 100 mL/min with carotid Doppler recording (i.e., the PC) and the remainder of each bag continued at any rate with no Doppler recording (i.e., after the PC). If instead, PLR was chosen as the PC, then the patient would have also received 2 PLRs (1 prior to the 1-L bag and 1 prior to the 500-mL bag) with carotid Doppler recording only during the PLR (i.e., the PC) but not during fluid administration (i.e., after the PC).

Prior to and immediately after each PC, vital signs were documented, as well, the total time required for each bag of IV fluid was recorded. The total time for each bag began with the onset of the RFC (or the beginning of the infusion after the PLR) and ended when the entire bag of fluid was administered. Only IV fluid administered for resuscitation was considered in this analysis (i.e., fluid given by gravity or maximal pump infusion rate), maintenance fluids were not included.

The primary outcome measure was the fraction of fluid unresponsive patients early in ED care and, therefore, total volume of ‘ineffective’ IV fluid (IVF_ineff_) administered to these patients. The secondary outcome measure was the time spent in the ED delivering IVF_ineff_, per patient. IVF_ineff_ was defined as any volume of fluid administered for which the antecedent PC disclosed an absolute ccFT_∆_ of less than + 7 ms. This was the optimal threshold for detecting a 10% SV_∆_ as identified by Barjaktarevic and colleagues [22].

Additional analyses were the same as above, however, different thresholds of ccFT_∆_ were used to define IVF_ineff_. For instance, in the investigation by Barjaktarevic et al. a + 4 ms absolute ccFT_∆_ was as accurate as + 7 ms, however, + 4 ms traded improved sensitivity for specificity. Further, in previous research on healthy volunteers performing a simple preload modifying maneuver [20] or undergoing moderate-to-severe central hypovolemia and simulated blood transfusion [21, 31], we found that 2% and 4% ccFT_∆_, respectively, best identified a 10% SV_∆_. Hence, we studied these thresholds as well.

As exploratory analyses, the change in heart rate (HR_∆_), in beats per minute (bpm), and mean arterial pressure (MAP_∆_), in millimeters of mercury (mmHg) as calculated from immediately before to immediately after the preload challenge were compared via a 2-tailed Student’s t-test between ‘effective’ and ‘ineffective’ preload challenges as determined for the different ccFT_∆_ thresholds listed above, but only if determined to be normally distributed by Kolmogorov–Smirnov testing. We tested the null hypothesis that there is no difference between the HR_∆_ (or MAP_∆_) between ineffective and effective preload challenges. Lastly, using Chi-squared we tested the relationship between ‘effective’ and ‘ineffective’ preload challenges and the clinician’s use of an RFC or PLR.

## Results

53 patients were enrolled for study and 2 were excluded because no usable Doppler spectra could be obtained. The clinical characteristics of the 51 patients included in this analysis are summarized in Table 1. Across the 51 patients, 94 PCs were performed and 8 PCs were excluded because there was no clear dicrotic notch discernable on the carotid spectrogram to calculate ccFT; therefore, 86 total PCs are included in this investigation comprising 19,667 carotid Doppler beats. 14% of patients were admitted to the ICU; 63% of patients were admitted to the general medical or surgical floor and 20% of patients were discharged from the ED. 17% had norepinephrine initiated in the emergency department and the 28-day re-admission and mortality rates for the 51 patients were 24% and 10%, respectively.

**Table 1:** Baseline patient characteristics

Number of patients	51
Female sex, *n* (%)	26 (51%)
Age, mean (SD)	64 (17.1)
Body mass index (SD)	28.7 (7.4)
IV fluids prior to enrollment (mL/kg), mean (SD)	1.6 (3.0)
Comorbidities, *n* (%)	
Congestive heart failure	8 (16%)
Ejection fraction*(%), mean	57%
Chronic kidney disease	9 (18%)
Chronic obstructive pulmonary disease	10 (20%)
Diabetes	11 (22%)
Cirrhosis	1 (2%)
Malignancy	5 (10%)
Immunosuppressed**	12 (24%)
Presentation	
Infection or sepsis suspected, *n* (%)	38 (75%)
Heart rate (beats per minute), mean (SD)	101.4 (22.5)
Mean arterial pressure (mmHg), mean (SD)	81.2 (19.4)
Temperature (^o^F), mean (SD)	37.4 (1.0)
Oxygen saturation (%), mean (SD)	96 (3)
Corrected flow time (ms), mean (SD)	299.4 (35)
Indication for IV fluids, *n* (%)	
Hypotension	36 (71%)
Tachycardia	33 (65%)
Low urine output	1 (2%)
Acute kidney injury	13 (25%)
Elevated lactate	10 (20%)
Confusion	7 (14%)
Other	36 (71%)

*Ejection fraction within 12 months of ED presentation, if available; based on 25 patients with this data

**Defined as active malignancy, AIDS or receiving immunosuppressive medications

**Fig. 2:**
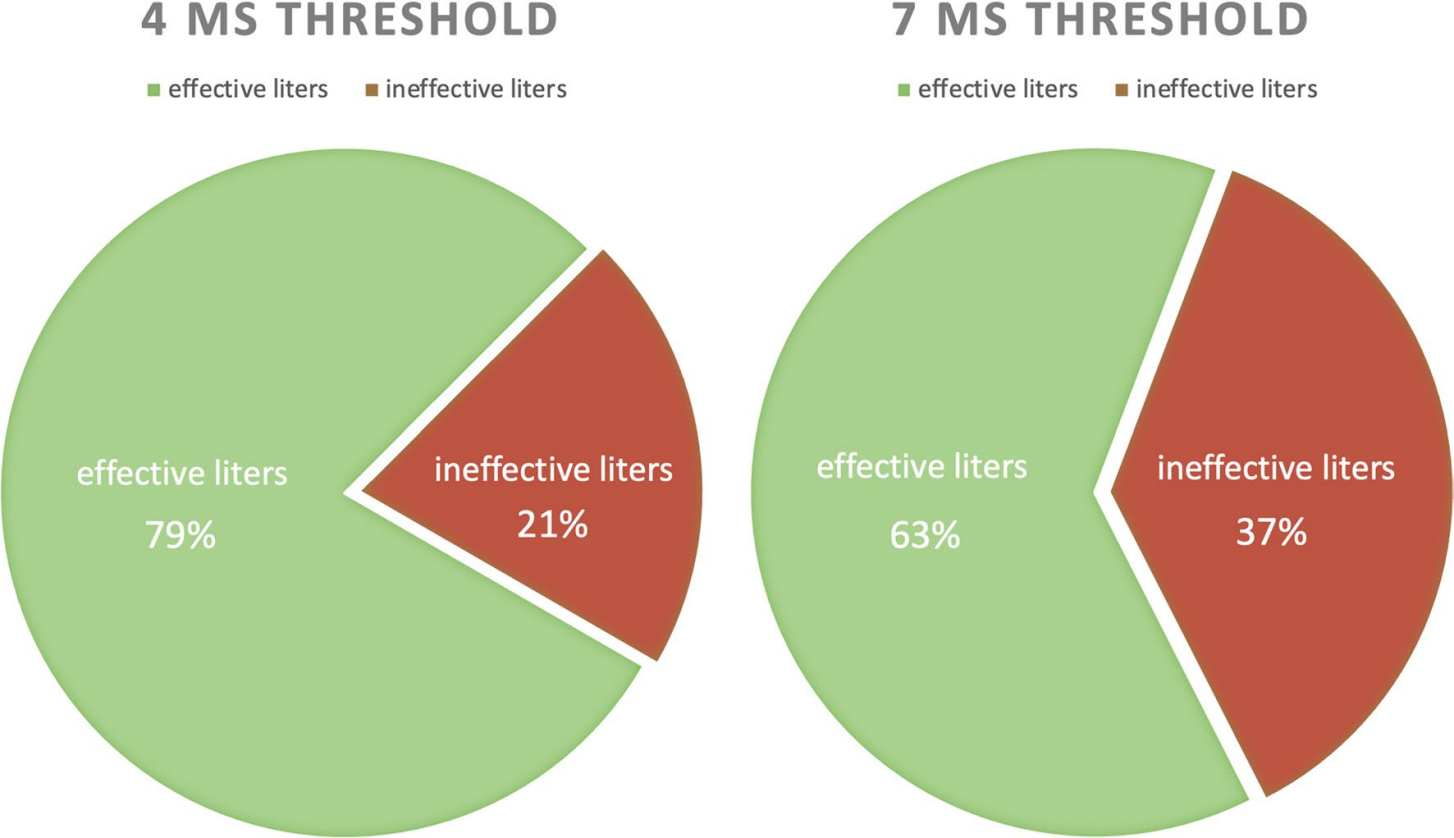
Distribution of effective and ineffective preload challenges based on ccFT_∆_ thresholds determined by Barjaktarevic and colleagues

Of the 86 PCs, 77 (90%) were via RFC and, of these, 65 (84%) were achieved via the LifeFlow device. In total, 81.7 L of IV fluid were delivered to the 51 patients (i.e., a mean of 1.6 L/patient); the average infusion duration was 67 min/L, or 97.8 min per patient, on average. The distribution of these 81.7 L into ‘effective’ or ‘ineffective’ based upon different ccFT thresholds is shown in Fig. 2. In Table 2, we report the fraction of unresponsive patients on first assessment, during any assessment or on all assessments as determined by different, previously reported, optimal ccFT thresholds.

**Table 2:** The fraction of unresponsive patients and total time burden of ineffective fluids based on different ccFT thresholds

ccFT_∆_ threshold	1st assessment unresponsive (%)	Any assessment unresponsive (%)	Always unresponsive (%)	IVF_ineff_ total (L)	IVF_ineff_ per patient (L)	Duration IVF_ineff_ per patient (min)
+ 7.0 ms [20]	41	53	29	30.0 L	0.6 L	35 min
+ 4.0 ms [20]	24	29	18	17.0 L	0.3 L	16 min
+ 2% [21]	37	49	25	27.5 L	0.5 L	32 min
+ 4% [30]	57	80	51	51.5 L	1.0 L	61 min

The relationship between change in HR (HR_∆_, in bpm) and change in MAP (MAP_∆_, in mmHg) before and after a PC for ‘effective’ and ‘ineffective’ assessments is shown in Table 3. Both HR and MAP were normal in distribution by Kolmogorov–Smirnov testing. There was no statistically significant change in HR or MAP between effective and ineffective PCs as defined by any threshold.

**Table 3:** The change in heart rate and mean arterial pressure between effective and ineffective preload challenges as determined by different ccFT thresholds

ccFT_∆_ threshold	Average HR_∆_ for ‘effective’ PC	Average HR_∆_ for ‘ineffective’ PC	*p*-value	Average MAP_∆_ for ‘effective’ PC	Average MAP_∆_ for ‘ineffective’ PC	*p*-value
+ 7.0 ms [20]	− 2.0	-3.0	0.4	1.3	0.8	0.9
+ 4.0 ms [20]	− 2.0	-3.0	0.3	0.7	2.9	0.7
+ 2% [21]	− 2.0	-3.0	0.09	1.3	0.7	0.4
+ 4% [30]	− 3.0	-2.0	0.3	2.1	0.5	0.8

With respect to the interaction between ‘effective’ and ‘ineffective’ PCs and the clinician’s decision to employ a rapid fluid challenge or passive leg raise, we found there to be no statistically significant interaction by Chi-squared testing; however, only 9 of the 86 PCs were by PLR.

## Discussion

In this pilot study conducted in a large, community ED, we enrolled patients requiring IV fluid administration based upon clinical examination. With respect to our primary goal, we observed a clinically significant fraction of fluid ‘unresponsive’ or ‘refractory’ [15] patients. This determination was based upon change in the carotid artery corrected flow time and was true whether a threshold with higher sensitivity (i.e., + 4 ms) or specificity (i.e., + 7 ms) was chosen [22]. A practical implication of these findings was measured in our secondary goal, that is, we observed a relatively large proportion of patients early in their care receiving physiologically ineffective IV fluids that consume valuable time in the ED. For instance, even with the + 4 ms threshold, our results indicate that for every 100 patients ordered IV fluid, approximately 28 h of ED care are spent on an intervention without its intended effect. As the 2021 operating expense of an ED with 100,000 annual visits ranges from $600–3000 per bed-hour (USD) [32], this represents $17,000 to $84,990 of hidden time cost; with the + 7 ms threshold, these totals approximately double for the same 100 patients.

As discussed above, the rates of fluid ‘unresponsiveness’ noted in this observational report are comparable to those observed by other investigators [10, 13, 16–18], however, our population was unselected, adult ED patients as compared to patients with sepsis and septic shock. Therefore, comparisons are not straightforward. For example, Kuan and colleagues used the PLR-induced change in bioreactance-measured stroke volume of at least 10% as the reference standard [16]. They observed a 28-day mortality rate of 9.8% and 21.3% of patients in their ED were unresponsive on first presentation. In our cohort, 75% of patients had infection-related hypoperfusion, the 28-day mortality rate was also 10% and, per the 4 ms ccFT_∆_ threshold, we noted an initial 24% unresponsiveness rate, comparable to Kuan et al. [16]. As well, in the ANDROMEDA-SHOCK investigation, which used pulse pressure variation in sedated, ventilated patients and PLR with change in Doppler-derived stroke volume in spontaneously breathing patients, approximately one-quarter of initial assessments were unresponsive, though in a much sicker, septic population [18]. Leisman and colleagues retrospectively determined the clinical response to fluid therapy in a large cohort of septic ED patients [33]. Importantly, in their study, they qualified the response to IV fluids based upon a patient achieving normotension without vasoactive medications—perhaps better termed ‘baro-responsive’ given that ‘fluid responsive’ typically connotes blood flow [3]. Further, they identified six clinical risk factors to predict which patients would remain hypotensive following fluids. Nevertheless, even in those without *any* clinical risk factors, they observed that more than one-in-four hypotensive, septic patients did not normalize their blood pressure with fluids alone. We suspect that the great majority of these low-risk patients did not augment SV with preload and, therefore, could have been detected earlier in their ED course with flow-guided monitoring.

Lastly, and from more of a clinical-physiological perspective, we appreciate that the + 7 ms threshold has a lower sensitivity (i.e., 70%) than specificity (i.e., 96%), meaning that those marked as fluid unresponsive are more likely to be a false negative than those above this threshold are to be a false positive [22]. This is why we also considered the + 4 ms threshold which, as reported by Barjaktarevic and colleagues [22], reduces the false negative rate by roughly 13%, though raises the false positive rate by a similar amount. Based on these observations, we wonder if different thresholds could be applicable in different treatment environments. For example, in clinical scenarios where under-resuscitation is deemed more concerning (e.g., early ED septic shock in a young patient), a lower, ‘fluid liberal’, ccFT_∆_ might be considered as a treatment threshold; whereas, in situations where over-resuscitation becomes more worrisome (e.g., later in ICU care in a patient with cardiovascular disease), a higher, ‘fluid conservative’, ccFT_∆_ threshold might be employed to dichotomize fluid non-responders from responders. Future, prospective evaluations wherein these thresholds are used to guide management could resolve which is clinically superior.

The primary limitation of this study is that we did not directly measure SV_∆_ as a reference standard for determining the physiological effect of IV fluid. It might be considered that the lack of change in heart rate and blood pressure between ‘effective’ and ‘ineffective’ preload challenges (Table 3) invalidates ccFT as a marker of SV_∆_. However, it has been known for decades that neither heart rate nor blood pressure can accurately gauge SV_∆_ [2, 3, 34]. Furthermore, ccFT is a promising surrogate for % SV_∆_ [35–43], even in the critically ill [22, 44], as mentioned in a recent systematic review [45]. Further, our data in healthy volunteers undergoing moderate-to-severe central hypovolemia, followed by simulated blood transfusion—induced by lower body negative pressure (LBNP) and release—demonstrated a strong, linear relationship between SV_∆_ and ccFT_∆_ [21, 31]. This LBNP analysis comprised approximately 50,000 cardiac cycles making it, to our knowledge, the largest known physiological comparison between SV_∆_ and ccFT_∆_. Nevertheless, a recent ED study found that ccFT_∆_ was unable to accurately detect a significant change in cardiac output [46]. As noted by the authors, this discrepancy may have been due to the skill-level of the sonographers performing the measurements. Further, we have observed clinically significant algorithm-lag between bioreactance SV_∆_ and Doppler ultrasonography [47, 48] and that respiratory cycle-mediated ccFT variability requires averaging at least 6–7 cardiac cycles (and often more) before and after an intervention to detect change with statistical confidence [30]. Therefore, timing with the reference standard must be closely regarded and many more than 1–3 cardiac cycles should be recorded in the carotid artery.

Second, we did not restrict our inclusion criteria to patients in shock or a specific etiology of hypoperfusion. Therefore, applying the ccFT_∆_ thresholds determined by Barjaktarevic and colleagues may not be physiologically appropriate given that their patient population was undifferentiated shock in the ICU [22]. Nevertheless, we applied various thresholds for detecting a 10% SV_∆_ including from healthy volunteers performing a simple preload modifying maneuver [20] and those in an LBNP chamber [31] which may more closely approximate the spectrum of hemodynamic duress in the ED. Despite this, for all thresholds studied, we observed a clinically significant fraction of ED patients, early in their care, demonstrating a fluid ‘unresponsive’ or ‘refractory’ phenotype. The physiological implication of these observations is unclear, but given that withholding IV fluids in much sicker, unresponsive patients is without adverse effect [9], we postulate that refraining from IV fluids in the unresponsive patients observed in this general ED population would also have been unharmful. IV fluid in these unresponsive patients could represent an unnecessary therapy and additional time cost for ED providers that could be safely stopped.

Third, 2 patients and 8 preload challenges were excluded. In the 2 excluded patients, both had grossly atypical carotid Doppler morphology with no clear dicrotic notch. As these patients may have had undisclosed cardiovascular disease, it is possible that excluding them from our analysis could bias our results and diminish generalizability. Nevertheless, the average age and baseline characteristics of our patient population are expected given our relatively broad inclusion criteria within a community ED. Further, the non-selective, pragmatic, inclusion criteria could be considered beneficial given that it reflects typical IV bolus practice in the ED.

Finally, the total time burden of ineffective IV fluid was determined not just by the fraction of patients who received physiologically ineffective fluid, but also the rate at which the fluid was administered. We asked the treating clinicians not to deviate from their typical care which improves the external validity of our observational study. However, if each bag of fluid was administered as rapidly as the RFC, then the total time spent would have been less. The average duration of slightly over 1 h per litre reflects the common practice of running IV fluid at 999 mL/h based on maximal pump speed.

## Conclusions

In conclusion, using both sensitive and specific ccFT_∆_ thresholds, we observed a clinically significant fraction of fluid ‘unresponsive’ or ‘refractory’ patients early in their ED care. This could represent cryptic patient and time cost in the ED. Given that there is a movement to treat IV fluids akin to antibiotics (i.e., ‘fluid stewardship’ [49]), administering IV fluid to refractory patients would be comparable to treating an infection with antibiotics to which the offending micro-organism is resistant—a course of action that is unhelpful, time-consuming and worthy of practice improvement. Future investigation will better define the role of early, flow-guided care in the ED, especially with regard to patient population selection, decision support, outcome, but also ED provider time-utilization, throughput and care-cost. Towards these ends, a lightweight, wireless, wearable Doppler ultrasound may establish new areas of investigation and practice within the emergency department and intensive care unit.

## Data Availability

The datasets used and/or analyzed during the current study are available from the corresponding author on reasonable request.

## Abbreviations

IV: Intravenous
SV: Stroke volume
ED: Emergency department
ICU: Intensive care unit
ccFT_∆_: Change in the carotid artery corrected flow time
mL: Milliliters
ccFT: Carotid artery corrected flow time
PC: Preload challenge
PLR: Passive leg raise
L: Liter
IVF_ineff_: Ineffective IV fluids
ms: Milliseconds
SV_∆_: Change in stroke volume
HR: Heart rate
MAP: Mean arterial pressure
mmHg: Millimeters of mercury
HR_∆_: Change in heart rate
MAP_∆_: Change in mean arterial pressure
